# Ingroup favoritism overrides fairness when resources are limited

**DOI:** 10.1038/s41598-022-08460-1

**Published:** 2022-03-16

**Authors:** Jihwan Chae, Kunil Kim, Yuri Kim, Gahyun Lim, Daeeun Kim, Hackjin Kim

**Affiliations:** Laboratory of Social and Decision Neuroscience, School of Psychology, Korea University, 145 Anam-ro, Seongbuk-gu, Seoul, 02841 Republic of Korea

**Keywords:** Psychology, Human behaviour

## Abstract

Ingroup favoritism and fairness are two potentially competing motives guiding intergroup behaviors in human. Here, we investigate if and how limited resources can modulate the way these two motives affect individuals’ decisions in intergroup situation. In the present study, participants (*N* = 58) were asked to accept or reject three types of resource allocation proposals generated by a computer: the ingroup advantageous condition, outgroup advantageous condition, and neutral condition. In general, participants were more willing to accept the proposals in the ingroup advantageous condition than the outgroup advantageous or the neutral conditions, and also in the moderate inequality than the extreme inequality condition. This may indicate that people sought a careful balance between ingroup favoritism and fairness, although we also found marked individual differences in their preferences for ingroup favoritism or fairness. Importantly, as predicted, participants were more likely to show ingroup favoritism only when limited resources affect the well-being of ingroup members. The present study provides novel insights into the situational and personality factors affecting human intergroup behaviors, shedding light on motives underlying intergroup conflicts prevalent in human societies.

## Introduction

Ingroup support or ingroup favoritism refers to the tendency to favor ingroup over outgroup members and protect or support one’s group members^1,2^. Ingroup loyalty, which has been proposed as one of the five major moral foundations^3^, is found in the early stage of life^4^. For example, infants prefer food and objects provided by natives than by non-native speakers^5,6^, selectively imitate ingroup compared with outgroup members^7,8^, and generate ingroup-centered prejudice or bias even when merely assigned arbitrarily to a minimal group with little interaction among members^9^. This minimal group effect is generally found in adulthood as well^10,11^; indeed, ingroup favoritism can be generally found in every human interaction across all ages, cultures, and groups, although the level of favoritism may differ^12^.

Ingroup favoritism plays a pivotal role in human behavior, especially in intergroup interaction. While ingroup favoritism may cause intergroup conflict and tension^13,14^, it can also increase one’s care toward other ingroup members and thus increase survival probability at the group level^15^. Therefore, ingroup favoritism, deeply ingrained in humans, is a double-edged sword in terms of survival, as it causes both ingroup support and intergroup conflict at the same time^1,2^.

Unlike ingroup favoritism, fairness refers to impartial treatment and attitudes without any favoritism or discrimination^17^, and equality would be the central principle of fairness^16^. Fairness can lead to cooperation between individuals and groups as well as reduce potential conflict and tension^18,19^. Similar to ingroup favoritism, this sense of fairness appears to be deeply rooted in humans’ moral cognition^20–22^. Supporting this idea, studies have shown that even infants have a sense of fairness^23,24^.

Although both ingroup favoritism and fairness play key roles in intergroup dynamics^25,26^, these two main moral principles often contradict each other^1,17,25,27^, especially in intergroup interaction where support for ingroup members can require treating outgroup members unfairly. It should be noted, however, the conflict between these two principles may depend on contextual factors^16,28–32^. For example, competition in sports primes fairness as a predominant moral principle^29^. By contrast, when people are exposed to a threat cue, their psychological boundary between the ingroup and outgroup becomes salient and thus increases ingroup bias^32^.

Some previous studies showed that limited resource would induce intergroup conflict and increase ingroup bias^33–35^. For example, based on their mean-looking time, infants who generally expect fair splits between ingroup and outgroup members change to expect ingroup-biased allocation when resources are limited^34^. On the other hand, other studies reported decreased, rather than increased, ingroup bias due to limited resources^36,37^. For example, a field study with adults showed that seasonal scarcity decreased one’s ingroup bias compared to the abundance period^36^. One possible reason for the inconsistency among these studies is that the terms “scarcity” and “limited resources” were ambiguously defined.

**Figure 1:**
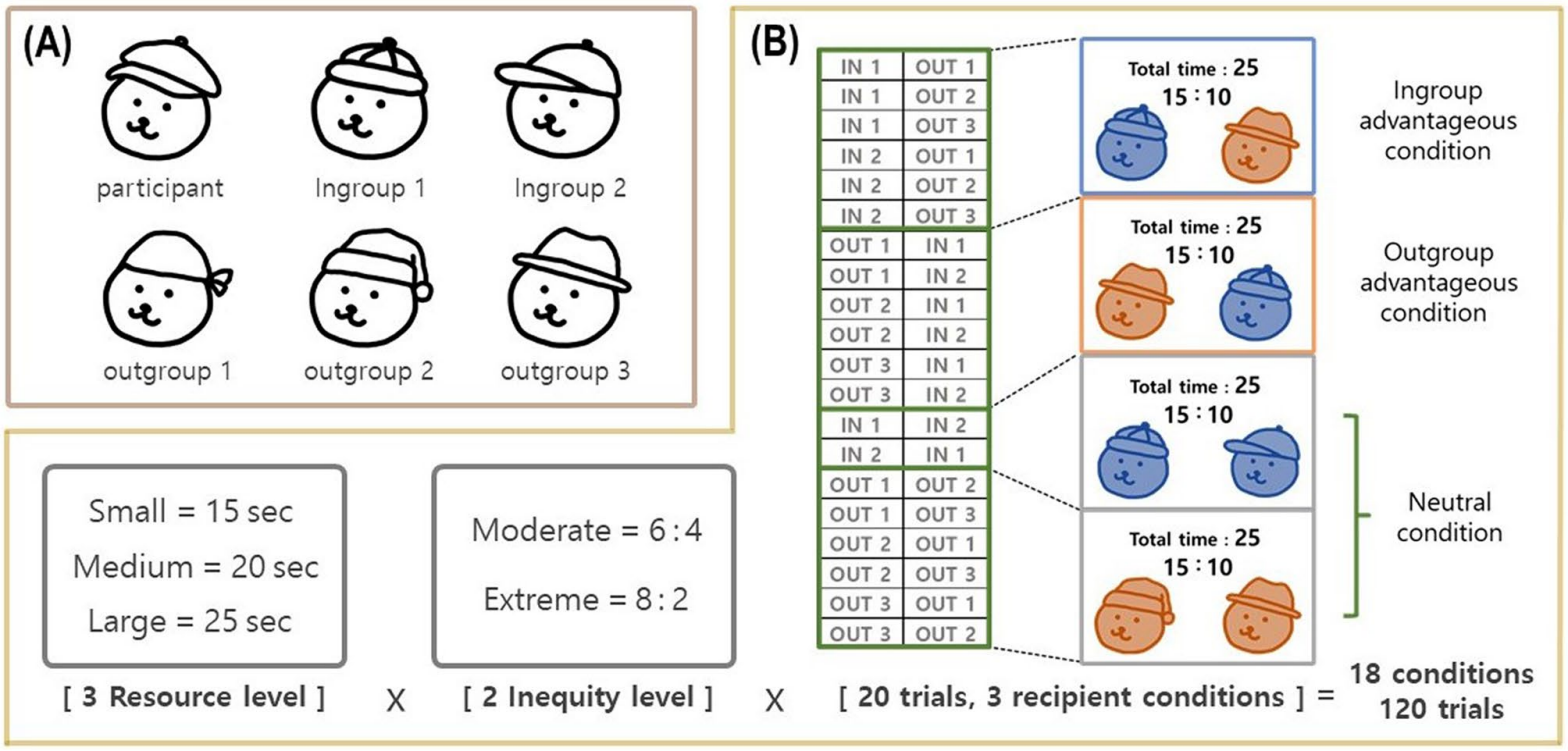
Schematic diagram of the experimental paradigm. (**A**) Six bear characters indicate the participants and the other five players. The red and blue colors were used to indicate their group membership (i.e., ingroup or outgroup), counterbalanced across participants. (**B**) The combination of three resource levels, two inequality levels, and three recipient types made up 18 conditions, with a total of 120 trials.

**Figure 2:**
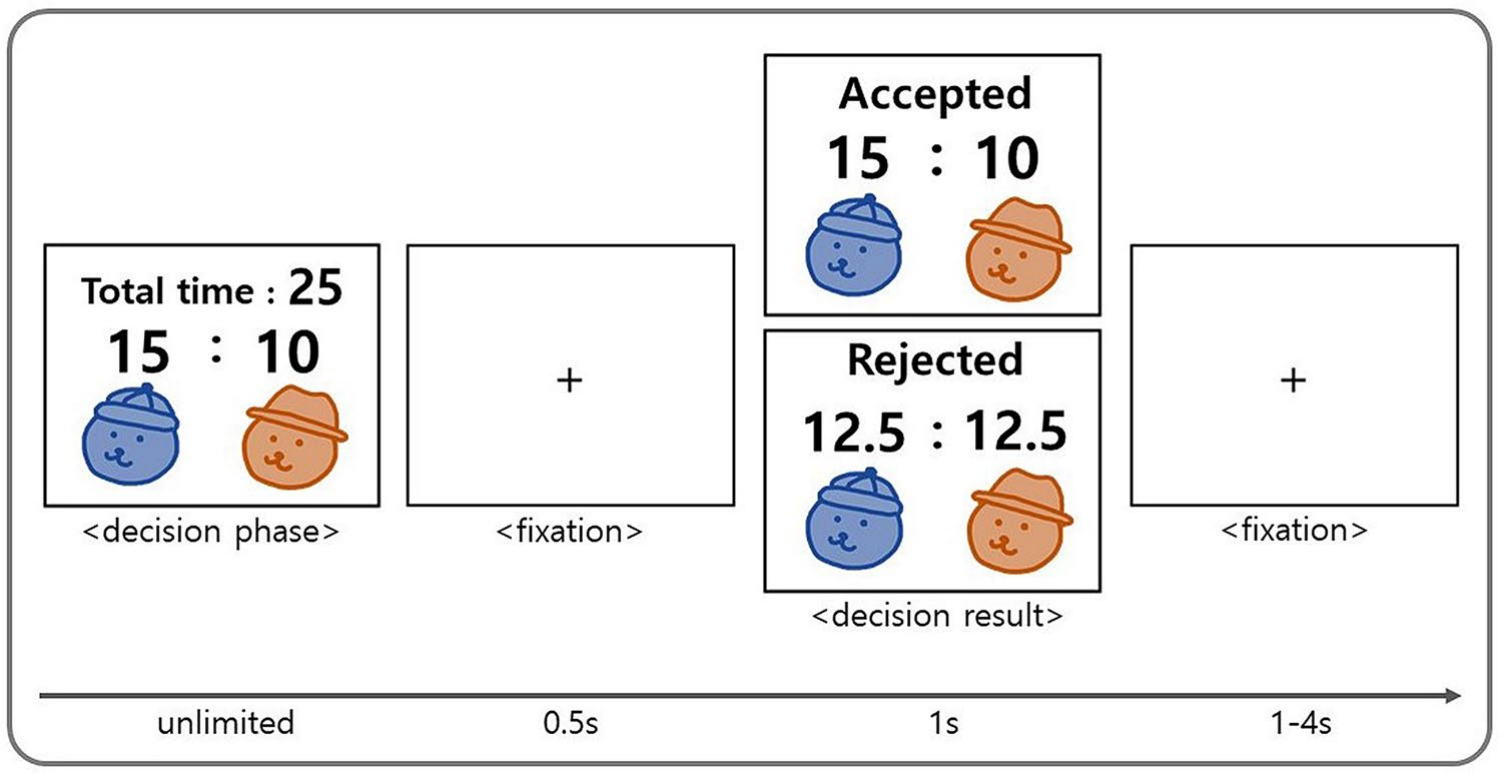
Schematic diagram of the BIDG. In the decision phase, a suggested allocation was displayed and participants were asked to choose to either accept or reject the suggestion. If they accepted, the time resource was distributed as suggested, whereas it was evenly distributed if rejected, and the decision result was displayed for 1 s following a fixation period of 0.5 s after the decision.

In the present study, we extend previous studies of the role of scarcity in modulating the conflict between ingroup favoritism and inequality aversion. First, we recruited adult participants for an economic game to assess whether we can generalize the findings of Bian et al.^34^ who only inferred implicit attitudes based on infants’ mean looking time. Second, we systematically varied the resource level including levels sufficient or insufficient to serve both the ingroup and the outgroup when distributed equally, to distinguish between limited and merely smaller resources. For this purpose, we developed a novel version of the third-party binary intergroup dictator game (BIDG). In the BIDG, the participant became the third party belonging to one of the two participating groups and decide the time split for the two recipients randomly chosen from the two groups (Fig. 1A, 1B; see the Materials and Method section for further detail). In each trial of the task, the suggestion of the time distribution to two recipients was presented on the screen, and participants could either accept or reject this suggestion. If accepted, the time resource would be distributed as suggested, whereas it would be evenly distributed if rejected (Fig. 2). Participants were told that the distributed time was then used as the time limit for the recipients to solve a math problem and recipients could earn or lose one game point for a correct or an incorrect answer, respectively. Participants were also told that the distributed time following each decision would only influence the one corresponding math problem and would not carry over to the other trials.

The distribution suggestion consisted of (1) the total amount of time to be distributed (i.e., resource amount), (2) the suggested distribution (i.e., inequality level), and (3) to which team participants allocated more time (i.e., recipient type) (Fig. 1B). The amount of time consisted of three levels: small (i.e., 15 s), medium (i.e., 20 s), and large (i.e., 25 s). The distribution ratio or inequality level consisted of moderate (i.e., 6:4) and extreme (i.e., 8:2) levels. The distribution suggestion for each recipient was shown on the screen. For example, if the resource amount was 25 s (large) and the inequality level was 8:2, then [20 s: 5 s] was shown on the screen as the suggested distribution. There were 20 pairs of recipients, with each pair randomly selected from their five counterparts; one of each pair was chosen to be the advantageous recipient and the other served as the disadvantageous recipient. In the ingroup advantageous condition, an ingroup member was selected to be the advantageous recipient and an outgroup member served as the disadvantageous recipient; the pairing was reversed for the outgroup advantageous condition. In some of the trials, both recipients were selected from the same group (i.e., ingroup-ingroup trials and outgroup-outgroup trials), which was named the neutral condition. These 20 pairs were presented equally in each of the six conditions of the three resource levels intersected with the two inequality levels, which resulted in 120 trials (Fig. 1B).

Before the main experiment, participants were instructed that each math problem takes about 10 s to solve on average, which would allow them to infer that rejecting the suggestion (i.e., fair distribution) could lead to an insufficient resource allocation being provided to either of the recipients in some trials. For example, a fair distribution in the small resource amount condition (i.e., 15 s) would cause each recipient to receive only 7.5 s, which is below the average time needed (i.e., 10 s). Thus, the small resource condition represents the limited resource since a fair choice could undermine ingroup support. By contrast, the fair choice does not severely undermine the probability that both recipients solve the math problem in the medium (i.e., 20 s) and large (i.e., 25 s) conditions because a fair choice would give 10 s and 12.5 s to each recipient in these conditions, respectively. Moreover, 20 s in the medium condition is shorter than the 25 s in the large condition but may not be a limited resource, which was defined as an amount of resource that would undermine ingroup support when split fairly.

To rule out any unwanted strategic motives behind participants’ choices in the BIDG, we provided the following two instructions. First, participants were told that none of their five counterparts would know that the time resources had been distributed by participants; therefore, their decision as the distributor would be anonymous to all recipients. Second, participants were told that they would distribute the time as a third party and would not receive any personal benefits based on their decisions.

## Results

### Resource allocation decision

The BIDG included 18 conditions with combinations of three resource amounts (small, medium, and large), two inequality levels (moderate and extreme), and three recipient types (advantageous, disadvantageous, and neutral). We then calculated the average acceptance rates for each of these 18 conditions (Supplementary Table S1 for the descriptive statistics) and entered them into a three-way rmANOVA with resource amount, inequality level, and recipient type as within-subject factors.

**Figure 3:**
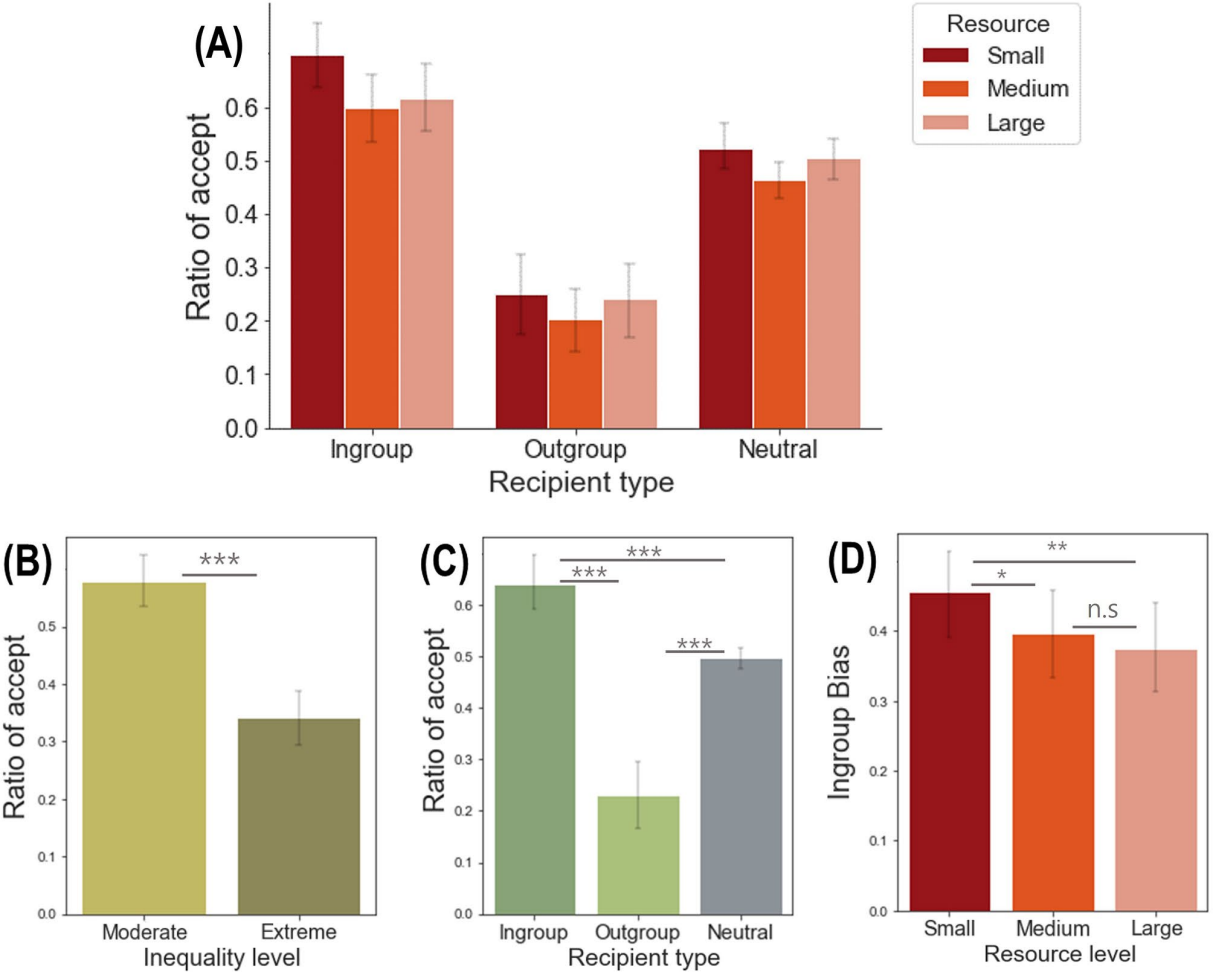
Behavioral results. (**A**) There was a significant interaction effect between resource level and recipient type but the main effect of resource level was only marginally significant. (**B**) Participants accepted more in the moderate condition than in the extreme condition. (**C**) Participants accepted more in the ingroup advantageous condition than the outgroup advantageous condition or the neutral condition. (**D**) The ingroup bias score in the small resource level was significantly higher than those in the medium and large conditions, although the difference between the small and medium conditions did not survive a Bonferroni correction. All error bars indicate the within-subject 95% confidence interval^46^. *p < 0.05, **p < 0.01, ***p < 0.001.

The rmANOVA with a Greenhouse–Geisser correction showed that the main effect of resource amount on the acceptance rate was only marginally significant, *F*(1.277, 72.791) = 3.601, *p* = 0.052, *η*_*p*_^2^ = 0.059 (Fig. 3A; Supplementary Table S2 and S3 for further detail). The main effect of inequality level was significant, *F*(1, 57) = 26.572, *p* < 0.001, *η*_*p*_^2^ = 0.318. Participants accepted the suggestion more in the moderate condition than the extreme condition, implying that people are aversive to inequality at the group level (Fig. 3B). The main effect of recipient type on the acceptance rate with the Greenhouse–Geisser correction was significant as well, *F*(1.166, 66.462) = 47.234, *p* < 0.001, *η*_*p*_^2^ = 0.453 (Fig. 3C). We conducted a post-hoc analysis based on the Bonferroni correction and thus the level of significance was adjusted to *p* < 0.05/3 = 0.016. The results showed that participants accepted the suggestions more in the ingroup advantageous condition (*M* = 0.64, *SD* = 0.31) than the outgroup advantageous condition (*M* = 0.23, *SD* = 0.23), *t*(57) = 7.125, *p* < 0.001, Cohen’s *d* = 0.94, implying that people generally prefer to support ingroup members than outgroup members. The acceptance rate in the neutral condition was lower than that in the ingroup advantageous condition, *t*(57) = −5.442, *p* < 0.001, Cohen’s *d* = 0.71, but higher than that in the outgroup advantageous condition, *t*(57) = 6.888, *p* < 0.001, Cohen’s *d* = 0.91.

The three-way interaction was not significant, *F*(4, 228) = 0.453, *p* = 0.770, *η*_*p*_^2^ = 0.008. However, as hypothesized, there was a significant interaction effect between resource level and recipient type, *F*(4, 228) = 3.381, *p* = 0.010, *η*_*p*_^2^ = 0.056 (Fig. 3A). Since the main goal of the present study was to examine the effect of limited resources on ingroup bias, we conducted an additional one-way rmANOVA on the ingroup bias parameters (i.e., subtracting the acceptance rate in the outgroup advantageous condition from that in the ingroup advantageous conditions, which indicates the difference in acceptance rate due to group information.) with resource amount as the within-subject factor. The results showed the significant effect of resource amount on the ingroup bias parameters, *F*(2, 144) = 5.717, *p* = 0.004, *η*_*p*_^2^ = 0.091 (Fig. 3D). We conducted the post-hoc comparisons using the Bonferroni correction and the level of significance was adjusted to *p* < 0.05/3 = 0.016. The results showed that the ingroup bias parameter was higher in the small condition (*M* = 0.45, *SD* = 0.44) than the large condition (*M* = 0.38, *SD* = 0.45), *t*(57) = 2.899, *p* = 0.005, Cohen’s *d* = 0.38. Although it did not survive the more conservative adjusted *p*-value, there was a significant difference between the small and medium conditions (*M* = 0.40, *SD* = 0.15), *t*(57) = 2.401, *p* = 0.020, Cohen’s *d* = 0.32. No significant difference was found between the medium and large conditions, *t*(57) = 1.038, *p* = 0.30, Cohen’s *d* = 0.14. Taken together, in line with our initial hypothesis, these results showed that ingroup bias increases when resources are limited (i.e., small as opposed to medium and large conditions), although merely a smaller resource (i.e., medium vs. large condition) does not influence ingroup bias at the group level.

### Response time

To analyze the RT data, we first detected and removed the outlier trials using median absolute deviation methods^38^. Then, we standardized the within-subject RT data by transforming them into Z-scores. Similar to the resource allocation decision analysis, we conducted a rmANOVA on the standardized RTs. The results showed that the main effect of resource amount on RT was not significant, *F*(2, 144) = 1.361, *p* = 0.260, *η*_*p*_^2^ = 0.023, whereas the main effect of inequality level was significant *F*(1, 57) = 37.659, *p* < 0.001, *η*_*p*_^2^ = 0.398 because participants showed a slower RT in the moderate condition (*M* = 0.15, *SD* = 0.18) than the extreme condition (*M* = −0.13, *SD* = 0.17) (Supplementary Sect. 3 for further discussion). The Greenhouse–Geisser-corrected result showed that the main effect of recipient type on RT was significant as well, *F*(1.753, 99.921) = 8.986, *p* < 0.001, *η*_*p*_^2^ = 0.136. The post-hoc comparisons with the Bonferroni-corrected *p*-value (*p* < 0.05/3 = 0.016) indicated that participants’ responses were significantly slower in the neutral condition (*M* = 0.01, *SD* = 0.21) than the ingroup advantageous condition (*M* = −0.04, *SD* = 0.20), *t*(57) = 3.039, *p* = 0.004, Cohen’s *d* = 0.40, and the outgroup advantageous condition (*M* = −0.07, *SD* = 0.18), *t*(57) = 3.750, *p* < 0.001, Cohen’s *d* = 0.50. However, no significant difference was found in the RT score between the ingroup and outgroup advantageous conditions. Taken together, participants showed a slower RT when both recipients are from the same group compared to when only one of the two recipients is ingroup.

### Correlation analysis

#### Correlation between the survey and main parameters

**Figure 4:**
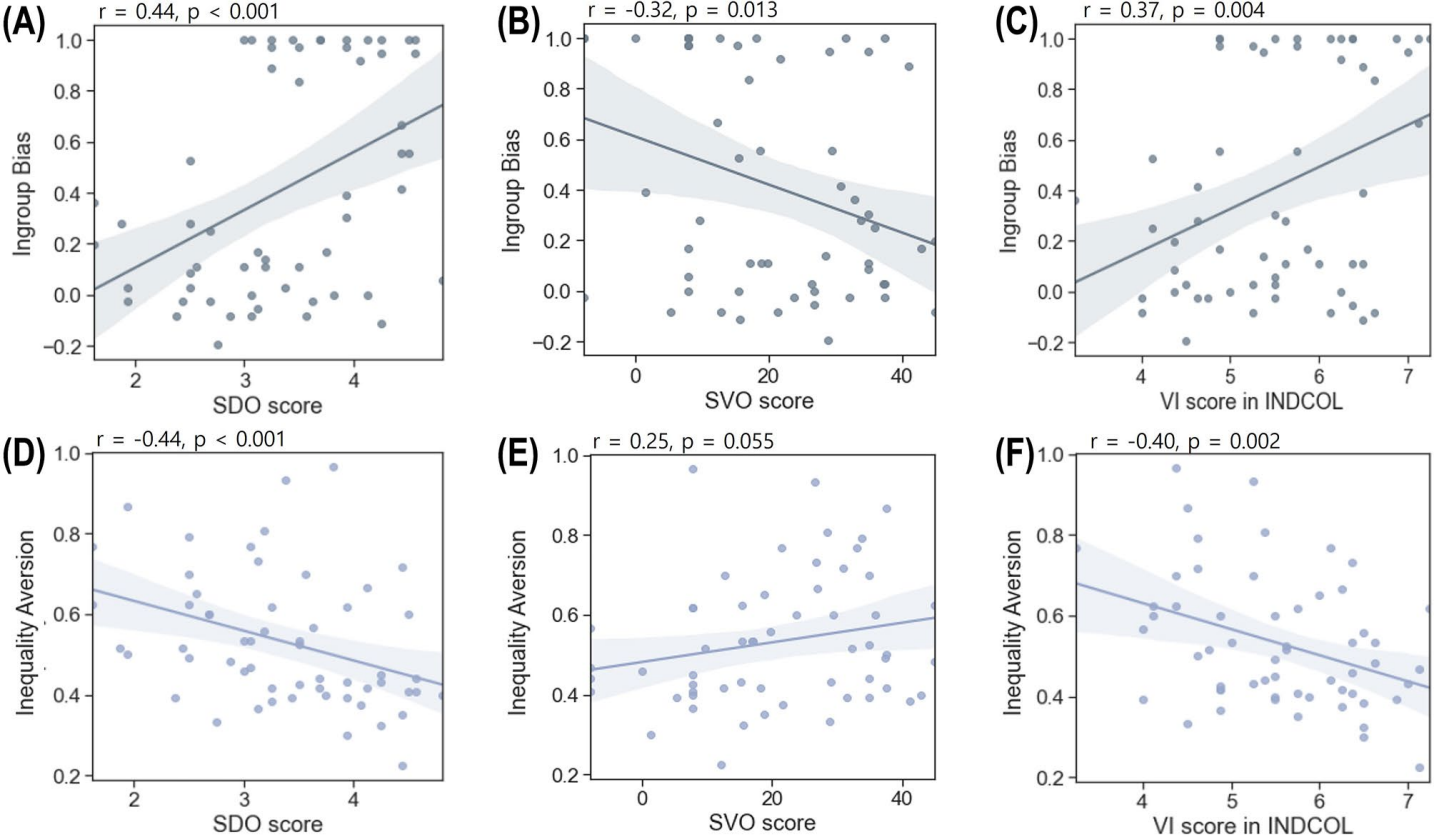
Individual differences in personality traits predicting ingroup bias and inequality aversion measured by the BIDG. (**A**) Individuals with higher ingroup bias parameters showed higher SDO scores, (**B**) lower SVO scores, and (**C**) higher VI scores. (**D**) Individuals with higher inequality aversion parameters showed lower SDO scores, (**E**) higher SVO scores with marginal significance, and (**F**) lower VI scores. the results are based on two-tailed Pearson correlation analyses and the line shadow indicates the 95% confidence interval.

We examined the relationships between the individual differences in the ingroup bias and inequality aversion parameters (i.e., overall reject rate, which indicates a preference for equal split over the suggested unequal split.) from the BIDG data and personality traits such as SDO, SVO, and INDCOL scores. A higher SVO score indicate higher prosocial tendency, and a higher SDO score indicate higher tendency of supporting social hierarchy and ingroup superiority. INDCOL consists of four dimensions and corresponding subscale scores such as vertical individualism (VI), vertical collectivism (CI), horizontal individualism (HI), and horizontal collectivism (HC). A higher score in each of the subscale scores would indicate higher tendency of its corresponding self-construal (Supplementary Sect. 1 for more information). The ingroup bias parameter correlated positively with the SDO scores, *r*(56)= 0.44, *p* < 0.001 (Fig. 4A), and negatively with the SVO scores, *r*(56) = −0.32, *p* = 0.013 (Fig. 4B). We also examined the relationships between the ingroup bias parameter and INDCOL score. Without any specific *a priori* hypotheses, we applied the adjusted *p*-value with Bonferroni correction (*p* < 0.05/4 = 0.0125). The results showed that the ingroup bias parameter was significantly correlated with VI, *r*(56) = 0.37, *p* = 0.004 (Fig. 4C), but not with any of the other subscale scores of INDCOL. By contrast, inequality aversion parameter correlated negatively with the SDO scores, *r*(56) = −0.44, *p* < 0.001 (Fig. 4D), and showed a marginal positive correlation with the SVO scores, *r*(56) = 0.25, *p* = 0.055 (Fig. 4E). As with the ingroup bias parameter, we applied the adjusted p-value with Bonferroni correction (*p* < 0.05/4 = 0.0125) for the correlation between Inequality aversion and INDCOL. The result showed that only the VI subscale score of INDCOL showed a significantly negative correlation with the inequality aversion parameters, *r*(56) = −0.40, *p* = 0.002 (Fig. 4F), and not the other subscale scores of INDCOL.

#### Correlations among the main parameters and RT scores

We conducted exploratory correlation analyses among the main parameters and RT data from the BIDG, which included seven variables: the ingroup bias parameter, inequality aversion parameter, RT in the ingroup condition, RT in the outgroup condition, RT in the neutral condition, RT in the extreme condition, and RT in the moderate condition. Without any specific *a priori* hypotheses, we applied the adjusted *p*-value with the Bonferroni correction (*p* < 0.05/21 = 0.0023).

**Figure 5:**
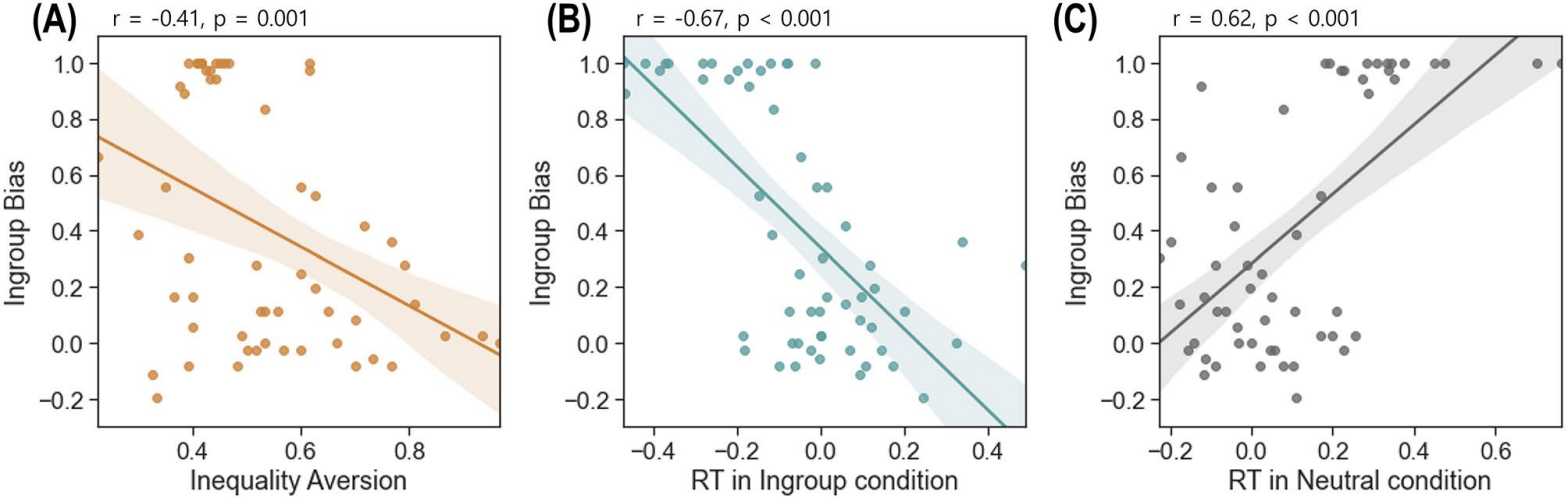
Correlations of the behavioral parameters with each other and RT. (**A**) The individual ingroup bias parameters were negatively correlated with the inequality aversion parameters. (**B**) Individuals with higher ingroup bias parameters showed faster RTs in the ingroup advantageous condition, (**C**) but slower RTs in the neutral condition. All the results are based on two-tailed Pearson correlation analyses and the line shadow indicates the 95% confidence interval.

As hypothesized, individuals’ ingroup bias parameter was negatively correlated with the inequality aversion parameter, *r*(56) = −0.41, *p* = 0.001. Interestingly, participants with a high ingroup bias parameter responded faster in the ingroup advantageous condition, *r*(56) = −0.67, *p* < 0.001 (Fig. 5B), and slower in the neutral condition, *r*(56) = 0.62, *p* < 0.001 (Fig. 5C). In addition, individual RT in the neutral condition was negatively correlated with RT in the ingroup advantageous condition, *r*(56) = −0.73, *p* < 0.001, and RT in the outgroup advantageous condition, *r*(56) = −0.64, *p* < 0.001. On the contrary, the individual RT scores in the extreme and moderate conditions were not correlated with any other parameters.

### Neutral condition analysis

**Figure 6:**
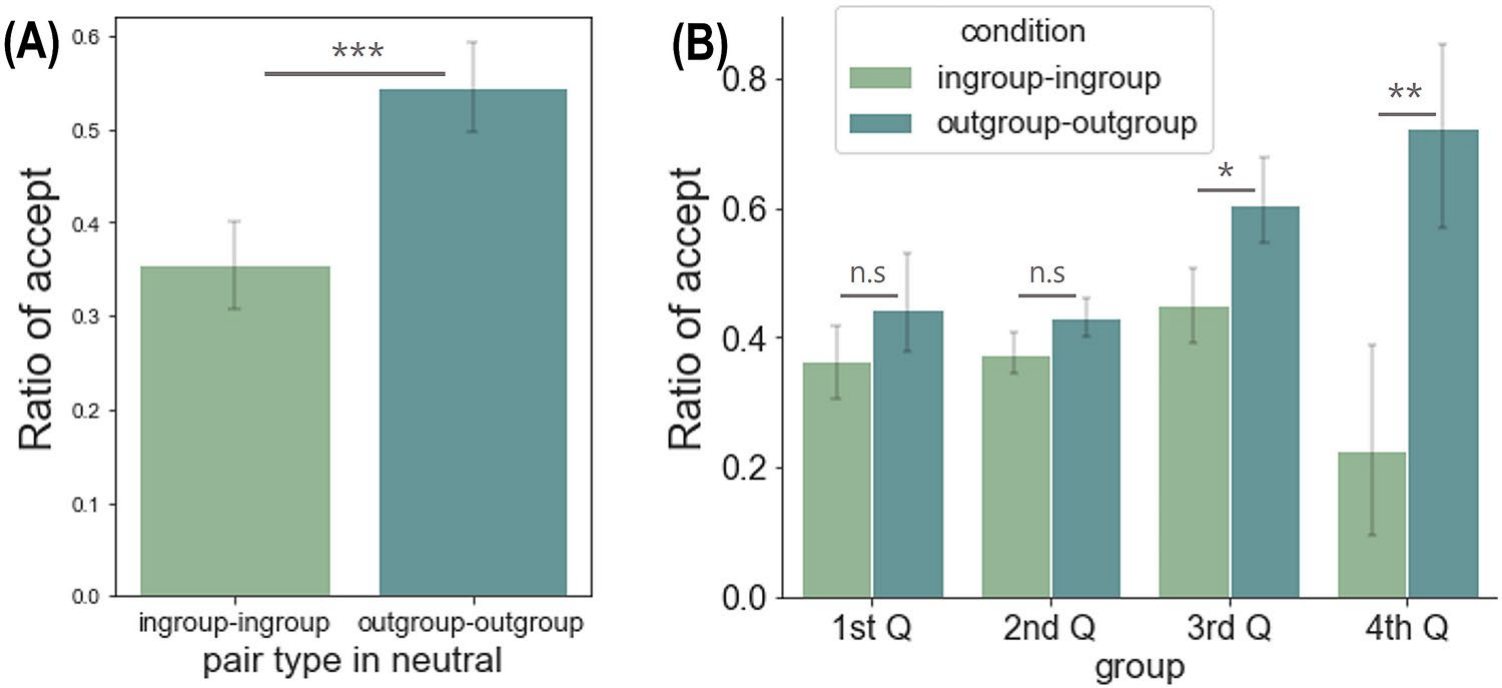
The results from the additional analyses for the neutral condition. (**A**) At the group level, participants accepted more in the outgroup-outgroup neutral condition than the ingroup-ingroup neutral condition. (**B**) We divided participants into four groups based on their sensitivity to the ingroup bias. The first group consisted of those with the lowest ingroup bias whereas the fourth group consisted of the highest. The differences in acceptance rate between each condition were mainly driven by the fourth and third quartile groups. The error bars indicate the within-subject 95% confidence interval^46^. *p < 0.05, **p < 0.01, ***p < 0.001.

The neutral condition consisted of two subconditions: the ingroup-ingroup and outgroup-outgroup conditions. We examined any differences between the two conditions in terms of the acceptance rate and RT. The results showed a significant difference in the acceptance rate between the ingroup-ingroup condition (*M* = 0.36, *SD* = 0.24) and outgroup-outgroup condition (*M* = 0.54, *SD* = 0.27), *t*(57)= − 3.780, *p* < 0.001, Cohen’s *d* = 0.50 (Fig. 6A). However, no differences were shown in the RT between the ingroup-ingroup condition (*M* = 0.08, *SD* = 0.33) and outgroup-outgroup condition (*M* = 0.08, *SD* = 0.24), *t*(57) = 0.073, *p* = 0.942, Cohen’s *d* = 0.01.

Given that the ingroup-ingroup condition differed from the outgroup-outgroup condition only in the group identity, we hypothesized that the difference in the acceptance rate between these two conditions is largely driven by those with higher sensitivity to group identity. Supporting this hypothesis, the ingroup bias parameter showed a significant positive correlation with the acceptance rate in the outgroup-outgroup condition, *r*(56) = 0.58, *p* < 0.001, along with a marginal negative correlation with that in the ingroup-ingroup condition, *r*(56)= −0.24, *p* = 0.073.

Based on these correlation results, we divided participants into four quartile groups by the ingroup bias score to test whether the participants with higher ingroup bias showed selective inequality aversion in the neutral condition. The first quartile group indicates participants with the lowest ingroup bias whereas the fourth quartile group indicates participants with the highest ingroup bias. We performed two-way mixed ANOVA with pair type (ingroup-ingroup pair and outgroup-outgroup pair) as within-subject factors and the quartile group (four-level) as between factors. Confirming our prediction, this analysis yielded a significant two-way interaction effect on the acceptance rate, *F*(3, 54) = 9.789, *p* < 0.001, *η*_*p*_^2^ = 0.352. We also found the main effects for the pair type, *F*(1, 54) = 20.269, *p* < 0.001, *η*_*p*_^2^ = 0.273, but not for the quartile group, *F*(3, 54) = 1.918, *p* = 0.138, *η*_*p*_^2^ = 0.096 (Fig. 6B). In addition, we conducted paired-sample t-test between two pair types in every quartile group. Results showed a significant difference in acceptance rate between the two pair types in the fourth quartile group, *t*(14) = 3.913, *p* = 0.002, Cohen’s *d* = 1.37, and the third quartile group, *t*(13) = 2.398, *p* = 0.032, Cohen’s *d* = 0.48, but not in the second quartile, *t*(13) = 1.706, *p* = 0.112, Cohen’s *d* = 0.21, and the first quartile group, *t*(14) = −0.696, *p* = 0.498, Cohen’s *d* = 0.07 (Supplementary Table S4 for descriptive statistics).

## Discussion

In the present study, we investigated how limited resources can modulate one’s moral preference and thus affect resource allocation decisions in intergroup situations. When there was a conflict between ingroup support (ingroup bias) and inequality aversion, participants were asked to accept or reject resource allocation proposals arbitrarily generated by a computer in three experimental conditions: the ingroup advantageous condition, outgroup advantageous condition, and neutral condition. In general, participants were more willing to accept an unfair proposal in the ingroup advantageous condition than in the outgroup advantageous or the neutral condition, while being more so in the moderate inequality condition than the extreme inequality condition. This indicated that they were motivated by both ingroup favoritism and inequality aversion, although there was a wide range of individual differences in their preference of either being ingroup supportive or being fair. Importantly, confirming our prediction, they were more likely to show ingroup favoritism when the available time resources were limited.

Previous studies of the effects of limited resources or scarcity on intergroup decisions have shown conflicting results^33–37^, presumably because “limited resources” and “scarcity” were arbitrarily determined in these studies and therefore varied. In the present study, we therefore systematically varied the resource amount and defined “scarcity” as a resource amount insufficient to serve both ingroup and outgroup members when resources are evenly distributed. This definition allowed us to differentiate limited resources from merely smaller resources and therefore test more accurately the hypothesis that ingroup bias overrides fairness when resources are limited.

Consistent with our prediction, ingroup bias was larger in the small condition than the medium and large conditions, whereas there was no significant difference between the medium and large conditions. This may be because ingroup support would be severely compromised if a participant chose to be fair, especially in the ingroup advantageous small condition because 7.5 s as a fair split of 15 s would be insufficient for both recipients to solve a math problem that takes 10 s on average. In other words, resource scarcity, not merely a lower amount of a resource, enhances ingroup bias. These results are in line with those of a previous study^34^ that found that infants expected ingroup support in intergroup resource allocation when resources were limited, whereas they expected fairness with higher resource amounts. The present study extended that study by showing that such a modulatory effect of limited resources on ingroup bias can be generalized from the inferred attitudes of infants to the explicit behaviors of adults.

At the group level, participants accepted more in the ingroup than the outgroup advantageous condition, implying that people favor ingroup support in general. In addition, participants accepted more in the moderate than the extreme condition, implying that people also seek moderate inequality than the extreme inequality. Although people were motivated by both ingroup support and fairness concerns, there were significant differences in the extent of these two motives, which were estimated using two independent parameters: ingroup bias and inequality aversion parameter. The ingroup bias parameter measured the tendency of individuals to prefer inequality in favor of ingroup over outgroup members whereas the inequality aversion parameter measured the general tendency of individuals to avoid inequality. The ingroup bias parameter showed a negative correlation with the inequality aversion parameter, implying that participants are mainly driven by the more dominant moral motive among these two, which is in line with previous studies^1,17,25,27^. In a similar vein, participants with higher ingroup bias showed higher SDO scores, lower SVO scores, and higher VI scores in INDCOL, whereas those with higher inequality aversion showed the opposite pattern.

Participants showed a faster RT in the ingroup and outgroup advantageous conditions than the neutral condition, which is in line with the findings of Volz et al.^26^. In addition, more ingroup-biased people (i.e., individuals with a higher ingroup bias parameter) responded faster in the ingroup and outgroup advantageous conditions but slower in the neutral condition, suggesting that they adopted a strategy to use the group membership information of recipients. Ingroup bias could expedite their decisions when recipients are from different groups but interfere with their decisions when such information is no longer available (i.e., the neutral condition). In addition, we found differences between the two subtypes of the neutral condition, which was not our main interest before the study. Specifically, participants accepted more in the outgroup-outgroup than the ingroup-ingroup condition. Individuals with more ingroup bias accepted more in the outgroup-outgroup condition and rejected more in the ingroup-ingroup condition. Given that those with higher ingroup bias were less inequality aversive, it is interesting that they became more inequality aversive only when both recipients are from the same group. Those with higher ingroup bias appear to show selective inequality aversion only when both recipients are ingroup members.

Taken together, the current study suggests that there are individual differences in preference for one of two moral principles of ingroup loyalty and fairness. Specifically, those with higher ingroup bias make faster resource allocation decisions when only one of the two recipients are ingroup compared to when both are from the same group, probably because they rely mostly on the recipients’ group membership information. Ingroup bias parameter is negatively correlated with inequality aversion parameter, which was supported by the three survey measures (i.e., SDO, SVO, INDCOL) as well. While those with ingroup bias were less likely to be inequality aversive, they could be selectively fair to ingroup members. Most importantly, ingroup favoritism overrode fairness at a group level when the available resources become limited.

Several issues deserve further discussion for future study. First, we used three resource levels (i.e., Small, Medium, and Large condition) in this study and only Small condition was regarded as a condition with limited resource because the amount of resource in this condition is intended to be insufficient to serve both ingroup and outgroup members when the resource is evenly distributed. Future studies would be necessary to investigate the effect of resource level on allocation behavior by increasing the range of resource levels and diversifying the resource levels to see exactly at what level people begin to prefer ingroup favoritism to fairness as measured by their allocation decisions, to define the term ‘scarcity’ more accurately.

Second, cultural difference is another factor that should be noted. The degree to which individuals prefer either of the two moral principles may vary significantly across cultures. Notably, vertical individualism scores in the INDCOL were correlated positively with the ingroup bias parameter and negatively with the inequality aversion parameter. Vertical individualism score consists of the questionnaires such as ‘it annoys me when other people perform better than I do.’ It is highly related to one’s tendency of social comparison and the degree of it varies across the cultures^39,40^. Given that the current study was conducted in one specific culture with limited demographic profiles (i.e., University students in Seoul, Republic of Korea), future studies would be needed to replicate the present findings in other cultures as well.

Finally, other forms of fairness should be tested in the future. In the current study, we used the terms fairness and inequality aversion interchangeably. Because the participants were not informed of potentially relevant personal information (i.e., socioeconomic status, merit) about the five counterparts other than their group identities and hats, equal distribution was the fairest principle in the current study. However, other principles (e.g., equity, merit-based allocation) would be fairer in different contexts^16,41^. For example, in the case of a company’s CEO, it would be fair to give more salaries to those who have achieved great performance, which can be called merit-based allocation, rather than simply giving equal salary to all members. In the case of social welfare sector, however, it would be fair to allocate more resources to people in need than to those who live well, which can be called equity-based allocation. Future studies should investigate whether other types of fairness can also be overridden by ingroup favoritism as resources are limited.

In conclusion, the present study demonstrated that limited resources could modulate one’s moral preference and thus affect allocation strategies. Confirming our *a priori* prediction, when resources are limited, people are more likely to choose ingroup support over fairness, which comprises two main motives for humans’ resource allocation behavior. We believe that the present study provides novel insights into the situational and personality factors affecting human intergroup behaviors and can be further extended to neuroimaging studies to identify the neural mechanisms underlying the intergroup conflicts prevalent in human societies.

## Materials and methods

### Participants

Sixty-nine undergraduate or graduate students (35 women; mean age = 24.3, ranging from 19 to 30 years) were recruited through the Korea university students’ community website. All participants were East Asians (i.e., 68 Korean, 1 Chinese). Eleven participants were excluded from the analysis: eight participants for being suspicious of the experimental cover story, one for misunderstanding the instructions, one for data loss due to technical problems, and one for showing a mean response time (RT; i.e., 6.9 s) longer than three standard deviations (i.e., 0.8 s) above the grand mean (i.e., 1.57 s). The remaining 58 participants’ data (29 females; mean age = 24.24, ranging from 19 to 29 years) were analyzed. To determine the appropriate sample size for this study, we conducted an *a priori* power analysis with G*Power 3.1.9.7^42^. The effect size (*η*_*p*_^2^ = 0.08) was drawn from an experiment in a previous study of intergroup resource allocation using a repeated measures ANOVA (rmANOVA;^16^). The power analysis yielded that the required sample size at α = 0.05 with 95% power was N = 48, indicating that the sample size of this study was sufficient to detect a medium effect^42^. The study protocol was approved by the Institutional Review Board of Korea University; all participants provided informed consent in written form to participate before the experiment and were compensated with KRW 9,000 or KRW 10,000, roughly equivalent to USD 9 and USD 10, respectively. All methods were performed under the relevant guidelines and regulations.

### Stimuli

Six bear icons were assigned to participants and five other players to show their respective identities in the main task (Fig. 1A). To examine the possible confounding effect of differences in preferences among the icons on resource allocation decisions in the main task, we obtained the preference ratings of each bear icon using a five-point Likert scale ranging from 1 (very bad) to 5 (very good) from participants during the recruiting phase via an online survey (Supplementary Sect. 2 for the results).

### Experimental procedures

During the recruiting phase as well as at the beginning of the experiment, participants were told that six players assigned with unique bear characters would interact via an online game without meeting each other to prevent any possible confounding effects caused by real interactions (Fig. 1A). Participants were told that each player was in a separate experimental room; however, there were no players other than the participant during the experiment.

Participants filled out a brief online survey where they were shown a visually bistable image named the Rubin-vase, which can be perceived either as two faces facing each other or as a vase and were asked to report which object they saw in the image. Upon arrival, participants were given either a red or a blue bear sticker to be attached to their shoulder to indicate the team to which they belonged (Supplementary Fig. S1). Participants were told that all participants were classified into two groups based on the perceptual differences inferred by the Rubin-vase test results and that three participants from one group had been recruited for the red team and another three participants from the other group for the blue team. Altogether, 32 out of the 58 participants were assigned to the red team in this study.

**Figure 7:**
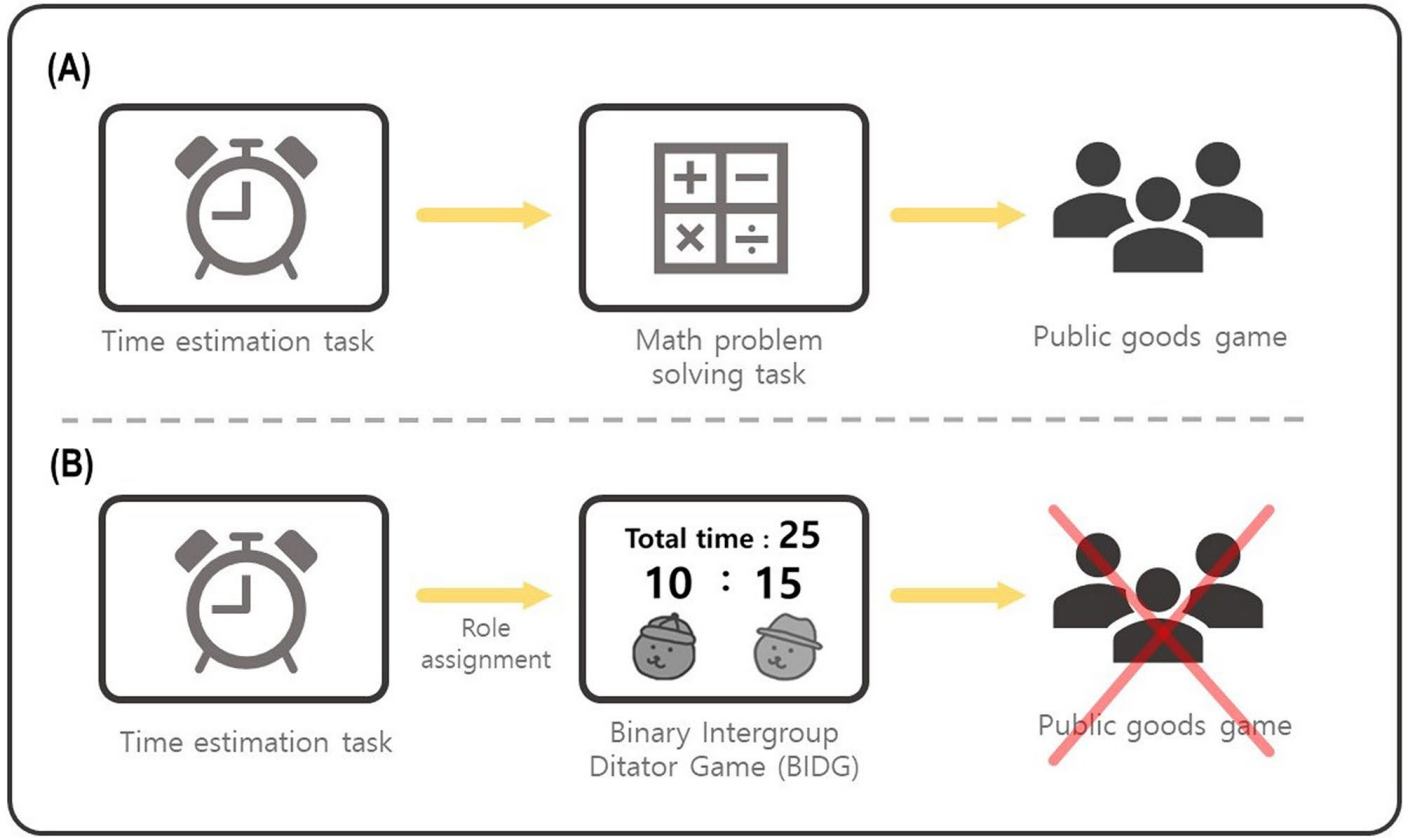
Schematic diagram of the experimental procedures. (**A**) In the cover story, participants were told that they would perform a time estimation task, math problem-solving task, and public goods game sequentially. (**B**) In reality, all participants were assigned to the role of the time distributor, performing the BIDG task instead, and the public goods games were not conducted.

Participants were instructed that they were to participate in three games in a row: a time estimation task, math problem-solving task, and public goods game. They were told that they would earn or lose the game point based on their performance in the first and second tasks and that the total game points earned would be used in the public goods game as a resource. In fact, the math problem-solving task and the public goods game were not conducted (Fig. 7). This instruction and game point were used to make participants believe that they were taking part in various types of related games and thus divert their attention from our main task (i.e., the BIDG) to avoid potential demand characteristics. They were told that different roles or conditions in the second game (i.e., the math problem-solving task) would be randomly assigned to them right after the end of the first game (i.e., the time estimation game). In fact, participants were always assigned to be the time distributor in the math problem-solving task, conducting the BIDG (Fig. 7B). In addition, participants were also told that each math problem takes about 10 s to solve an average, which was critical information when participants conducted the BIDG.

Participants performed the time estimation task as a team to increase their group identity (Supplementary Fig. S2). In this task, they were instructed that the target time (e.g., 3 s) would be presented on the monitor and that a beep would follow; they were also told that they should press the spacebar at the target time after the onset of the beep. The beep would then stop and the participant whose duration of the beep was closest to the target time would receive the highest score. In each trial of this task, they earned a game point if their team achieved a higher score than the other team did. In reality, the winning rate in this task was set to 50% to control for any confounding effect on their decisions in the subsequent BIDG task.

After the time estimation task, participants were assigned to the time distributor role and conducted the BIDG for the other five counterparts. They were to either accept or reject the time distribution suggestion for the two recipients randomly chosen from the five counterparts the BIDG consisted of 120 trials in total (Fig. 1B; Fig. 2; Supplementary Fig. S3).

After the BIDG, participants were debriefed that there were no counterparts and that the third game (i.e., public goods game) would not be conducted. Participants were asked whether they would provide consent for the data usage and whether they noticed any deceptions or demand characteristics in the study. Eight out of the 69 participants who replied that they did notice the non-existence of other participants were therefore excluded from the analyses.

After the experiment, participants completed three questionnaires on social value orientation (SVO^43^), social dominance orientation (SDO^44^), and individualism/collectivism (INDCOL^45^). These questionnaire data were used in the exploratory Pearson correlation analyses to examine the relationship between the self-report measure of personality traits and behavioral data obtained from the BIDG task.

### Parameters

#### Ingroup bias parameter

The acceptance rate of an unfair suggestion in the ingroup advantageous condition alone does not represent ingroup bias since participants might have accepted the unfair suggestions equally high (or equally low) in every condition. Therefore, we defined the ingroup bias parameter by subtracting the acceptance rate of an unfair suggestion in the outgroup advantageous condition from that in the ingroup advantageous condition, thereby reflecting the net difference in the acceptance rate of an unfair suggestion due to group identity. In sum, a higher score on this parameter would indicate a higher level of one’s ingroup bias.

#### Inequality aversion parameter

We used the overall reject rate (i.e., 1—overall acceptance rate) as the inequality aversion parameter as it could represent the level of individuals’ inequality aversion. Therefore, a higher score on this parameter would indicate a higher level of inequality aversion.

### Data acquisition and statistical analysis

We used PsychoPy version 2020.2.2 (https://www.psychopy.org, 2002–2018 Jonathan Peirce) to implement the behavioral tasks and acquire the behavioral data. Relevant data and code can be found at https://github.com/cog413/resource_distribution_experimient. Behavioral analyses (i.e., rmANOVA, paired-sample t-test, and Pearson correlation analyses) were performed using SPSS version 25.0 (IBM Corp., 2017) and Python version 3.8.3 (https://www.python.org, 2020). In rmANOVA, we reported p-values with standard degrees of freedom, but a Greenhouse–Geisser correction was applied in case the sphericity assumption was violated (Supplementary Table S2, S3). In all paired-sample t-test and Pearson correlation, we reported p-value based on a two-tailed test. Data visualization was performed via SPSS and the Python Matplotlib package (https://www.matplotlib.org, 2020).

## Supplementary Information

Supplementary Information.
