# Supplementary material for: Ingroup favoritism overrides fairness when resources are limited
Source: Sci Rep. 2022 Mar 16;12:4560. doi: 10.1038/s41598-022-08460-1 (PMC8927613; doi:10.1038/s41598-022-08460-1)
Supplement: Supplementary file 1 — Supplementary Information. [file 41598_2022_8460_MOESM1_ESM.docx]

**Ingroup favoritism overrides fairness
when resources are limited**

Jihwan Chae, Kunil Kim, Yuri Kim, Gahyun Lim, Daeeun Kim, and Hackjin Kim

Laboratory of Social and Decision Neuroscience
School of Psychology
Korea University, Seoul, Republic of Korea

***Correspondence**

Hackjin Kim, Ph.D.

School of Psychology

Korea University

145 Anam-ro, Seongbuk-gu, Seoul 136-701, Republic of Korea

hackjinkim@korea.ac.kr

**Section 1: Detailed description of the survey measures (i.e., SDO, SVO, and INDCOL)**

**Social Dominance Orientation:** SDO is a personality trait measuring an individual’s tendency of supporting social hierarchy and the extent to which they desire their in-group to be superior to out-groups ([1]). The SDO questionnaire is composed of 16 items with a 7-point Likert scale such as “some groups of people are simply inferior to other groups” and “We should do what we can to equalize conditions for different groups” (reverse-score). Individuals with a higher SDO score are likely to support their group to be superior. We therefore initially expected that the SDO score will be correlated positively with the ingroup bias parameter and negatively with inequality aversion parameter. Individual SDO score was calculated by averaging all responses, therefore ranging from 1 to 7 (Mean = 3.32, SD = 0.85).

**Social Value Orientation**: SVO is a questionnaire-based measure of equality preferences. It measures how much weight a person assigns to the welfare of others in relation to one’s own ([2]). SVO is related to many other social behaviors, especially cooperative decision-making ([3]). In this study, we used the SVO-slider measure with six items, each of which has nine point distributions between self and other. Originally, this measure calculates the angle with a range of -45 to 90 and categorize them into four groups which are competitor, individualist, prosocial, and altruist. As the higher angle in this measure indicates more prosocial tendency, we initially expected that the SVO score will be negatively correlated with the Ingroup Bias parameter as one would consider the outgroup’s welfare as well. On the other hand, we expected that the SVO score will be positively correlated with the Inequality Aversion parameter. The descriptive statistics in the study were as follows. [Mean = 20.99, Standard Deviation = 14.18].

**Individualism and Collectivism:** INDCOL measures one’s preference of either individualism or collectivism as well as one’s support for the hierarchy ([4]). A version of INDCOL with 32-items and 9-point Likert scale was used which is composed of the following four subscales:
- Vertical Individualism (VI): Measures the tendency of social comparison and the desire to outperform others (e.g., “It is important that I do my job better than others”).
- Horizontal Individualism (HI): Measures the tendency to be a unique and independent self, but not to compare themselves with others (e.g., “I’d rather depend on myself than others” ).
- Vertical Collectivism (VC): Measures the tendency to be submissive to their group and to the hierarchy within the group (e.g., “It is my duty to take care of my family, even when I have to sacrifice what I want”).
- Horizontal Collectivism (HC): Measures the tendency to be an equal and interdependent group member (e.g., “If a coworker gets a prize, I would feel proud”).
We didn’t have an initial hypothesis between the two main parameters (i.e., ingroup bias parameter, inequality aversion parameter) and the INDCOL score.

Responses for all the items in each subscale were averaged, which therefore ranged from 1 to 9. The descriptive statistics of each of the subscales were as follows. [VI: Mean = 5.51, Standard Deviation = 0.94; HI: Mean = 6.39, Standard Deviation = 0.94; VC: Mean = 6.09, Standard Deviation = 1.02; HC: Mean = 6.35, Standard Deviation = 1.01].

**Section 2: Preference rating and time distributed to each character**

To test the existence of the specific preference in the characters used as the symbols of other recipients, we measured the average preference rating for each character (see the Materials and Method section for further detail). We conducted a one-way rmANOVA for participants whose decision data were analyzed (N = 58). There was a significant difference in the preference for each character (*F*(5, 285) = 10.074, *p* < 0.001, $\eta_{p}^{2}$ = 0.150), indicating that this preference could affect the participant’s decision to allocate the resource unintentionally. This preference difference was mainly induced by the highest preference rating for the “outgroup 2” character (**Fig. 1A**).

To check whether the difference in preference affected the actual allocation decision, we calculated the average acceptance rate for each character presented as an advantageous recipient. No significant difference was found for the acceptance rate between the characters of the recipients within the same group. A paired-sample t-test between the two ingroup characters (“ingroup 1” and “ingroup 2”) showed no significant difference in the acceptance rate for either “ingroup 1” (*M* = 0.57, *SD* = 0.25) or “ingroup 2” (*M* = 0.56, *SD* = 0.25), *t*(57) = 1.266, *p* = 0.211, Cohen’s *d* = 0.17. The result of the one-way rmANOVA for the outgroup characters (“outgroup 1,” “outgroup 2,” and “outgroup 3”) also revealed no significant difference in the acceptance rate among the outgroup characters, *F*(2, 114) = 1.245, *p* < 0.292, $\eta_{p}^{2}$ = 0.021. Taken together, we found that character preferences did not affect the distribution decision.

**Section 3: Discussion of the RT differences in the Moderate condition and Extreme condition**

Participants responded slower in the moderate than in the extreme condition at the group level. However, the individual RT scores in the extreme and moderate conditions did not correlate with the behavioral parameters or any other survey scores. These results suggest that the difference in the RT seems to be mainly driven by the small value differences between the accept and reject options in the moderate condition other than the inequality aversion and ingroup favoritism. In the moderate condition, the value difference between the two options (i.e., 6:4 vs. 5:5) was smaller than that in the extreme condition (i.e., 8:2 vs. 5:5). Such small value differences in the moderate condition may have increased choice difficulty, thus leading to slower RTs ([5][6][7]).

**Section 4: Inequality sensitivity parameter**

We defined the inequality sensitivity parameter by subtracting the acceptance rate of an unfair suggestion in the extreme condition from that in the moderate condition, assuming that those who are sensitive to the inequality level would accept the unfair suggestion more in the moderate condition and less in the extreme condition to avoid an extremely unequal distribution. Therefore, a higher score on this parameter would indicate a higher level of one’s inequality sensitivity. Initially, we called this parameter as inequality aversion parameter. Owing to the reviewer’s comment, however, we realized that the overall rejection rate itself would better represent one’s inequality aversion. Therefore, we renamed this parameter as an inequality sensitivity parameter and report it as an additional parameter.

We conducted correlation analyses between the inequality aversion parameter and the survey scores (i.e., SDO, SVO, INDCOL). We hypothesized that the inequality sensitivity parameter would have a negative correlation with SDO score and a positive correlation with SVO score. As a result, inequality sensitivity parameter showed significant positive correlation with SDO score, *r*(56) = $-$0.37, *p* = 0.004, but not with the SVO score, *r*(56) = 0.18, *p* = 0.184. Without any specific *a priori* hypotheses, we applied the adjusted *p*-value with Bonferroni correction (*p* < 0.05/4 = 0.0125) for the INDCOL subscale scores. The inequality sensitivity parameter showed a significant negative correlation with VI, *r*(56) = $-$0.33, *p* = 0.012, but not with any of the other subscale scores of INDCOL.

Correlation analyses between the inequality parameter and the other parameters (i.e., ingroup bias parameter, inequality aversion parameter) were conducted as well. We initially hypothesized that the inequality sensitivity parameter would negatively correlate with the ingroup bias and positively correlate with the inequality aversion parameter. Ingroup bias parameter showed significantly positive correlation with inequality sensitivity parameter, *r*(56) = $-$0.52, *p* < 0.001. However, there were no correlation between inequality sensitivity parameter and inequality aversion parameter, *r*(56) = 0.21, *p* = 0.114.

| **Descriptive Statistics** | | | | | | | |
| --- | --- | --- | --- | --- | --- | --- | --- |
| **Recipient** | **Resource** | **Inequality** | **Mean** | **Std. Deviation** | **N** | **Min** | **Max** |
| Ingroup | Small | Moderate | 0.833 | 0.298 | 58 | 0 | 1 |
|  |  | Extreme | 0.563 | 0.438 | 58 | 0 | 1 |
|  | Medium | Moderate | 0.716 | 0.343 | 58 | 0 | 1 |
|  |  | Extreme | 0.480 | 0.444 | 58 | 0 | 1 |
|  | Large | Moderate | 0.756 | 0.323 | 58 | 0 | 1 |
|  |  | Extreme | 0.477 | 0.451 | 58 | 0 | 1 |
| Outgroup | Small | Moderate | 0.348 | 0.396 | 58 | 0 | 1 |
|  |  | Extreme | 0.152 | 0.288 | 58 | 0 | 1 |
|  | Meduim | Moderate | 0.307 | 0.350 | 58 | 0 | 1 |
|  |  | Extreme | 0.098 | 0.242 | 58 | 0 | 1 |
|  | Large | Moderate | 0.362 | 0.391 | 58 | 0 | 1 |
|  |  | Extreme | 0.121 | 0.237 | 58 | 0 | 1 |
| Neutral | Small | Moderate | 0.642 | 0.292 | 58 | 0 | 1 |
|  |  | Extreme | 0.403 | 0.343 | 58 | 0 | 1 |
|  | Meduim | Moderate | 0.571 | 0.311 | 58 | 0 | 1 |
|  |  | Extreme | 0.356 | 0.338 | 58 | 0 | 1 |
|  | Large | Moderate | 0.625 | 0.303 | 58 | 0 | 1 |
|  |  | Extreme | 0.384 | 0.348 | 58 | 0 | 1 |

**Supplementary Table S1 |** Descriptive statistics of individuals’ acceptance rates in each of the conditions.

| **Mauchly's Test of Sphericity^a^** | | | | | | | |
| --- | --- | --- | --- | --- | --- | --- | --- |
| **Within Subjects Effect** | **Mauchly's W** | **Approx. Chi-Square** | **df** | **Sig.** | **Epsilon^b^** | | |
|  |  |  |  |  | **Greenhouse-Geisser** | **Huynh-Feldt** | **Lower-bound** |
| resource | 0.434 | 46.761 | 2 | 0.000 | 0.639 | 0.647 | 0.500 |
| equity | 1.000 | 0.000 | 0 |  | 1.000 | 1.000 | 1.000 |
| recipient | 0.285 | 70.349 | 2 | 0.000 | 0.583 | 0.588 | 0.500 |
| resource * equity | 0.971 | 1.629 | 2 | 0.443 | 0.972 | 1.000 | 0.500 |
| resource * recipient | 0.747 | 16.130 | 9 | 0.064 | 0.891 | 0.958 | 0.250 |
| equity * recipient | 0.790 | 13.205 | 2 | 0.001 | 0.826 | 0.848 | 0.500 |
| resource * equity * recipient | 0.887 | 6.673 | 9 | 0.671 | 0.951 | 1.000 | 0.250 |
| Tests the null hypothesis that the error covariance matrix of the orthonormalized transformed dependent variables is proportional to an identity matrix. | | | | | | | |
| a. Design: Intercept   Within Subjects Design: resource + equity + recipient + resource * equity + resource * recipient + equity * recipient + resource * equity * recipient | | | | | | | |
| b. May be used to adjust the degrees of freedom for the averaged tests of significance. Corrected tests are displayed in the Tests of Within-Subjects Effects table. | | | | | | | |

**Supplementary Table S2 |** The table of the Mauchly’s Test of Sphericity for the rmANOVA. The condition which violated the sphericity assumption (i.e., with significance lower than 0.05) went through Greenhouse-Geisser correction and therefore was specifically mentioned when reporting the results.

| **Tests of Within-Subjects Effects** | | | | | | | |
| --- | --- | --- | --- | --- | --- | --- | --- |
| **Source** | | **Type III Sum of Squares** | **df** | **Mean Square** | **F** | **Sig.** | **Partial Eta Squared** |
| resource | Sphericity Assumed | 0.831 | 2 | 0.416 | 3.601 | 0.030 | 0.059 |
|  | Greenhouse-Geisser | 0.831 | 1.277 | 0.651 | 3.601 | 0.052 | 0.059 |
| Error(resource) | Sphericity Assumed | 13.157 | 114 | 0.115 |  |  |  |
|  | Greenhouse-Geisser | 13.157 | 72.791 | 0.181 |  |  |  |
| equity | Sphericity Assumed | 14.580 | 1 | 14.580 | 26.572 | 0.000 | 0.318 |
|  | Greenhouse-Geisser | 14.580 | 1.000 | 14.580 | 26.572 | 0.000 | 0.318 |
| Error(equity) | Sphericity Assumed | 31.275 | 57 | 0.549 |  |  |  |
|  | Greenhouse-Geisser | 31.275 | 57.000 | 0.549 |  |  |  |
| recipient | Sphericity Assumed | 29.603 | 2 | 14.801 | 47.234 | 0.000 | 0.453 |
|  | Greenhouse-Geisser | 29.603 | 1.166 | 25.388 | 47.234 | 0.000 | 0.453 |
| Error(recipient) | Sphericity Assumed | 35.724 | 114 | 0.313 |  |  |  |
|  | Greenhouse-Geisser | 35.724 | 66.462 | 0.538 |  |  |  |
| resource * equity | Sphericity Assumed | 0.049 | 2 | 0.025 | 0.789 | 0.457 | 0.014 |
|  | Greenhouse-Geisser | 0.049 | 1.944 | 0.025 | 0.789 | 0.454 | 0.014 |
| Error (resource*equity) | Sphericity Assumed | 3.551 | 114 | 0.031 |  |  |  |
|  | Greenhouse-Geisser | 3.551 | 110.822 | 0.032 |  |  |  |
| resource * recipient | Sphericity Assumed | 0.194 | 4 | 0.049 | 3.381 | 0.010 | 0.056 |
|  | Greenhouse-Geisser | 0.194 | 3.566 | 0.055 | 3.381 | 0.014 | 0.056 |
| Error (resource*recipient) | Sphericity Assumed | 3.277 | 228 | 0.014 |  |  |  |
|  | Greenhouse-Geisser | 3.277 | 203.261 | 0.016 |  |  |  |
| equity * recipient | Sphericity Assumed | 0.094 | 2 | 0.047 | 1.745 | 0.179 | 0.030 |
|  | Greenhouse-Geisser | 0.094 | 1.653 | 0.057 | 1.745 | 0.186 | 0.030 |
| Error (equity*recipient) | Sphericity Assumed | 3.084 | 114 | 0.027 |  |  |  |
|  | Greenhouse-Geisser | 3.084 | 94.210 | 0.033 |  |  |  |
| resource * equity * recipient | Sphericity Assumed | 0.025 | 4 | 0.006 | 0.453 | 0.770 | 0.008 |
|  | Greenhouse-Geisser | 0.025 | 3.805 | 0.007 | 0.453 | 0.761 | 0.008 |
| Error (resource*equity* recipient) | Sphericity Assumed | 3.153 | 228 | 0.014 |  |  |  |
|  | Greenhouse-Geisser | 3.153 | 216.864 | 0.015 |  |  |  |
| a. Computed using alpha = .05 | | | | | | | |

**Supplementary Table S3 |** The results of rmANOVA for the behavioral data (i.e.., mean acceptance rate for each condition).

| **Descriptive Statistics** | | | | | | | |
| --- | --- | --- | --- | --- | --- | --- | --- |
| group | **Ingroup bias score** | | **Acceptance rate in  ingroup-ingroup pair** | | **Acceptance rate in  outgroup-outgroup pair** | | N |
|  | Mean | Std. Deviation | Mean | Std. Deviation | Mean | Std. Deviation |  |
| 1 | -0.0537 | 0.0522 | 0.4056 | 0.2354 | 0.3852 | 0.1941 | 15 |
| 2 | 0.1131 | 0.0643 | 0.3750 | 0.1981 | 0.4306 | 0.1848 | 14 |
| 3 | 0.5655 | 0.2365 | 0.4524 | 0.2440 | 0.6052 | 0.2270 | 14 |
| 4 | 0.9907 | 0.0166 | 0.2056 | 0.2417 | 0.7481 | 0.3064 | 15 |
| Total | 0.4061 | 0.4304 | 0.3578 | 0.2438 | 0.5431 | 0.2712 | 58 |

**Supplementary Table S4 |** Descriptive statistics of the four quartile groups based on the ingroup bias score.


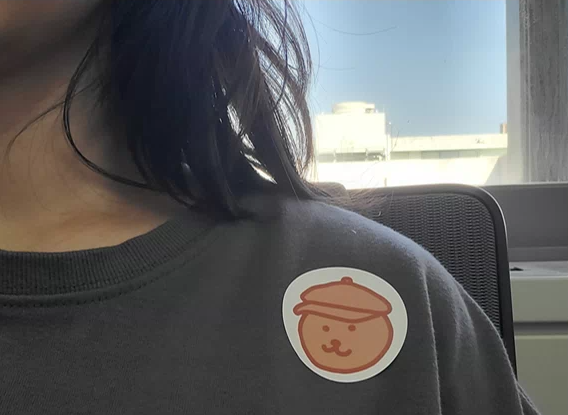


**Supplementary Fig. S1 |** An example bear sticker. Participants attached a bear sticker to their shoulder. The color of the bear indicated which team they belonged to. By attaching the sticker, the participant can be more immersed in their character and their team. 6 bear characters were designed by the 1st author.


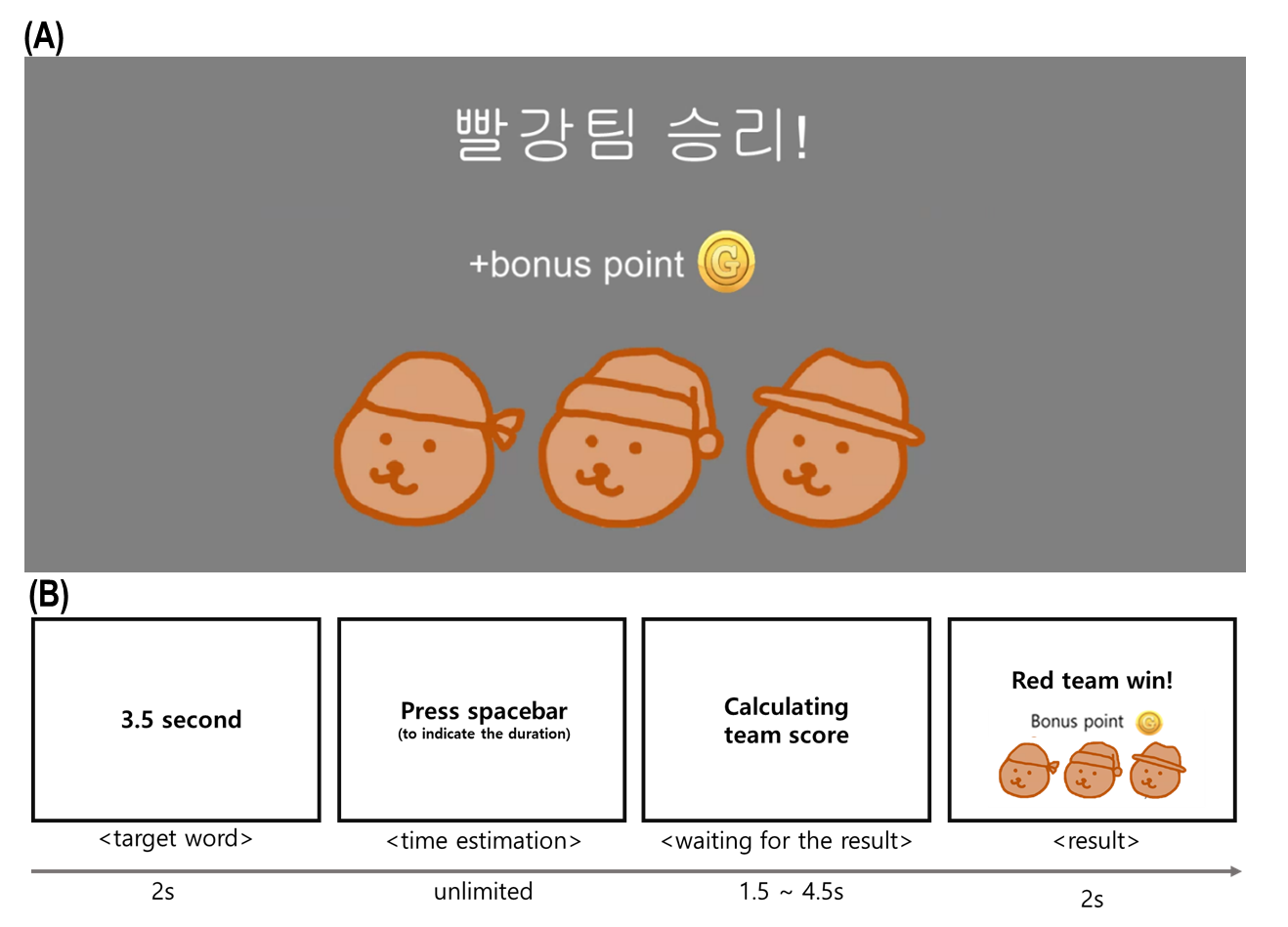


**Supplementary Fig. S2 |** An illustration of Time estimation task. (A) the actual task scene for the result. (B) the target time was presented on the monitor and the beep followed. Participants could press the spacebar to stop the beep. The duration of the beep was then used as the participant’s estimated target time. Participants were told that the closer estimation would earn the better score and the team with a higher total score would win the corresponding trial. In fact, the chance of winning or losing was controlled to 50:50.


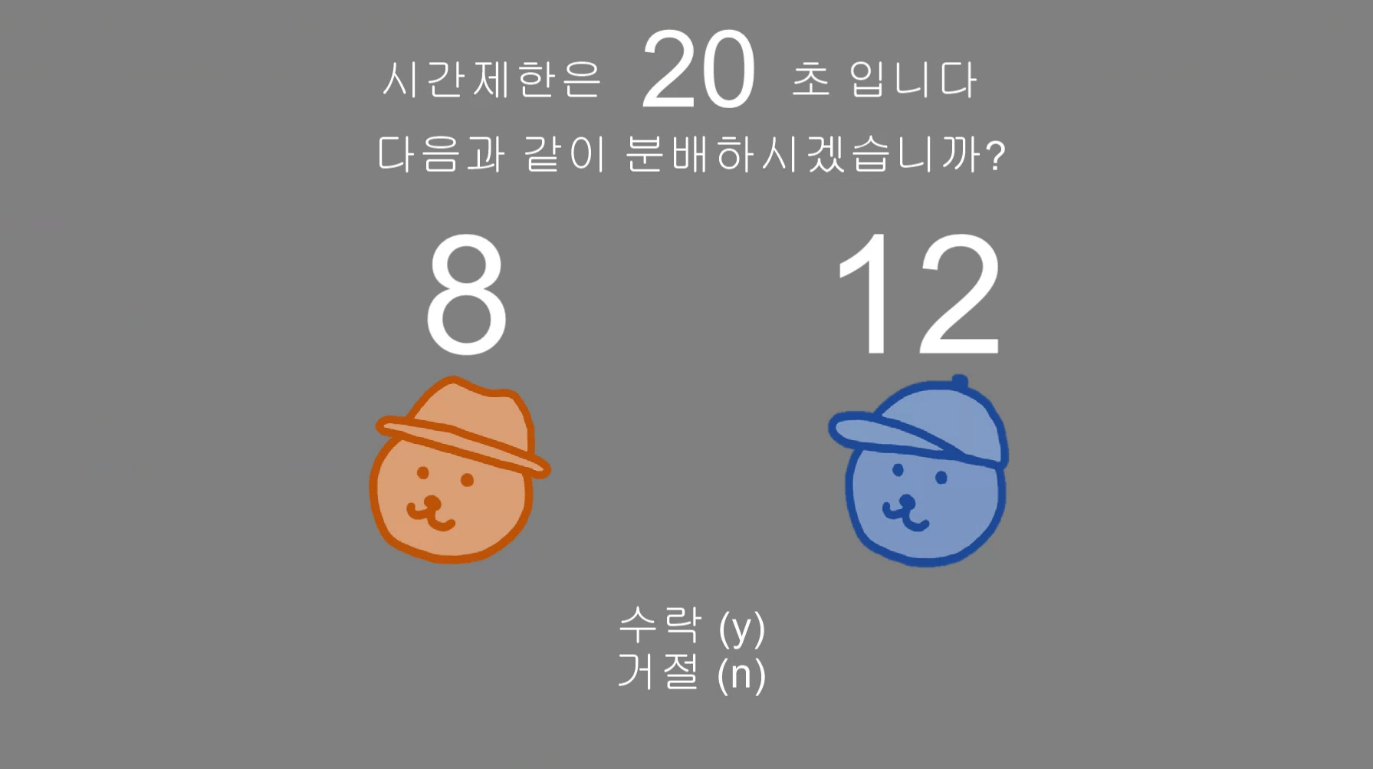


**Supplementary Fig. S3 |** The actual scene for the BIDG. Participants should press the ‘y’ key to accept the suggestion and the ‘n’ key to reject the suggestion. Since two characters (confederates) were presented horizontally to each other, we used vertically located keys (i.e., ‘y’ and ‘x’) to avoid any right or left positional bias.

**Supplementary References**

1. Pratto, F., Sidanius, J., Stallworth, L. M., & Malle, B. F. Social dominance orientation: A personality variable predicting social and political attitudes. *Journal of Personality and Social Psychology*, **67**, 741 (1994).
2. Murphy, R. O., Ackermann, K. A., & Handgraaf, M. Measuring social value orientation. *Judgment and Decision Making*, **6**, 771-781 (2011).
3. Bogaert, S., Boone, C., & Declerck, C. Social value orientation and cooperation in social dilemmas: A review and conceptual model. *British Journal of Social Psychology*, **47**, 453-480 (2008).
4. Triandis, H. C., & Gelfand, M. J. Converging measurement of horizontal and vertical individualism and collectivism. *Journal of Personality and Social Psychology*, **74**, 118. (1998).

Ratcliff, R., & Smith, P. L. A comparison of sequential sampling models for two-choice reaction time. *Psychological Review*, **111**, 333 (2004).

Shenhav, A., Straccia, M. A., Cohen, J. D., & Botvinick, M. M. Anterior cingulate engagement in a foraging context reflects choice difficulty, not foraging value. *Nature Neuroscience*, **17**, 1249-1254 (2014).

1. Vassena, E., Deraeve, J., & Alexander, W. H. Surprise, value and control in anterior cingulate cortex during speeded decision-making. *Nature Human Behaviour*, **4**, 412-422 (2020).
